# Multidimensional characteristics of musculoskeletal pain and risk of hip fractures among elderly adults: the first longitudinal evidence from CHARLS

**DOI:** 10.1186/s12891-023-07132-z

**Published:** 2024-01-02

**Authors:** Fengyao Mei, Jiao Jiao Li, Jianhao Lin, Dan Xing, Shengjie Dong

**Affiliations:** 1 Arthritis Clinic and Research Center, Peking University People’s Hospital, Beijing, 100044 P.R. China; 2 Thoracic surgery Department, Beijing Hospital, Beijing, 100044 China; 3 School of Biomedical Engineering, Faculty of Engineering and IT, University of Technology Sydney, Ultimo, NSW 2007 Australia; 4 Department of the Joint and Bone Surgery, Yantaishan Hospital, Yantai, China

**Keywords:** Musculoskeletal pain, Hip fractures, Risk factors, CHARLS, P for trend

## Abstract

**Background:**

Hip fractures are a major public health concern among middle-aged and older adults. It is important to understand the associated risk factors to inform health policies and develop better prevention strategies. Musculoskeletal pain is a possible implicating factor, being associated with physical inactivity and risk of falls. However, the association between musculoskeletal pain and hip fractures has not been clearly investigated.

**Methods:**

A nationally representative sample of the Chinese population was obtained from the China Health and Retirement Longitudinal Study (CHARLS). The study collected patient information on their demographic characteristics, socioeconomic status, other health-related behavior, and history of musculoskeletal pain and hip fractures. Univariate and multivariate analyses were conducted to investigate the factors influencing the risk of hip fracture, including factors related to the individual and to musculoskeletal pain. P for trend test was performed to assess the trend of each continuous variable. The robustness and bias were assessed using the bootstrap method. Restricted cubic spline regression was utilized to identify linear or non-linear relationships.

**Results:**

Among the 18,813 respondents, a total of 215 individuals reported that they have experienced a hip fracture. An increased risk of hip fracture was associated with the presence of waist pain and leg pain (*P* < 0.05), as well as with an increased number of musculoskeletal pain sites (*P* < 0.05). For individuals aged 65 and above, a significant association was found between age and the risk of hip fracture (*P* < 0.05). Furthermore, respondents with lower education level had a higher risk of hip fracture compared to those with higher education levels (*P* < 0.05).

**Conclusion:**

In the Chinese population, the risk of hip fracture was found to be associated with both the location and extent of musculoskeletal pain, as well as with other factors such as age and demographic characteristics. The findings of this study may be useful for informing policy development and treatment strategies, and provide evidence for comparison with data from other demographic populations.

**Supplementary Information:**

The online version contains supplementary material available at 10.1186/s12891-023-07132-z.

## Introduction

Musculoskeletal pain is a highly prevalent health issue, particularly among the elderly population, affecting up to 74% of older adults in global communities [1–3]. Musculoskeletal pain may be associated with prior injury or neuropathic causes, but in the majority of cases is idiopathic and as such requires long-term management [4]. Its chronic nature imposes a substantial burden on both individuals and society, leading to restricted physical function, diminished quality of life, and disability, which on the societal level result in significant loss of productivity and demand of healthcare resources [5]. The latest analysis of the Global Burden of Disease Study reported that in 2016, lower back pain ranked first and neck pain ranked sixth out of 30 major diseases and injuries contributing to years lived with disability [6]. Musculoskeletal pain commonly manifests in multiple anatomical sites. Various studies have reported that approximately 41–75% of individuals experience pain in two or more sites, although the prevalence may vary depending on the population studied and the number of pain sites assessed [3, 7, 8]. Epidemiological studies have demonstrated that compared to single-site pain, multi-site pain is linked to worse health outcomes including reduced physical function and health-related quality of life, impaired cognitive function and sleep quality, and heightened depressive symptoms [9–13].

Fractures pose a significant health concern globally, frequently resulting in morbidity and mortality particularly for elderly individuals, and can lead to heightened susceptibility to recurring fractures [14–16]. Significant risk factors for fractures include aging, osteoporosis, and falls [17]. Some associations of these same factors with musculoskeletal pain have been found. For instance, studies have revealed that experiencing musculoskeletal pain at particular sites within the body and also having multiple pain sites can separately increase the likelihood of falls [18, 19]. Furthermore, research has confirmed that having musculoskeletal pain in multiple sites is linked to the risk of fractures, even after adjusting for the risk of falls [20]. Among different types of fractures, hip fractures in particular pose a significant public health concern among individuals aged 60 and above, leading to untimely mortality, severe disability, and a decline in functional independence [21–23]. Patients who experience hip fractures are frequently at higher risk of complications such as cardiovascular and infectious diseases, bedsores, bleeding, and depression, which can ultimately result in death [24]. Recent studies have estimated the worldwide 1-year mortality rate for hip fractures at approximately 22%, with nearly 50% of survivors experiencing a loss of functional independence [25, 26]. The Burden of Disease Study in China identified hip fracture as a primary cause of years lived with disability among 32 different injury types [27].

Because hip fractures are associated with the socioeconomic consequences of chronic patient management and increased mortality in the elderly, it is important to gain a better understanding of the risk factors to inform health policies and develop prevention strategies. The nature of the primary patient population, consisting of older adults has posed certain barriers to investigation such as loss of follow-up. Recently, there was a greater emphasis on studying the prevention of hip fractures and exploring the related risk factors. However, there is still a lack of research on the association between musculoskeletal pain and hip fractures [28]. Current investigations of the relationship between musculoskeletal pain and fractures have been more focused on vertebral fractures, or reporting the incidence and cause of different types of fractures [29–32]. Limited studies have specifically investigated the association between musculoskeletal pain and hip fractures. In one study, Adren et al. reported that knee pain was a risk factor for hip fractures [33]. However, musculoskeletal pain often affects multiple sites, and the location of pain as well as multi-site compared to single-site pain may have different influences on the risk of hip fractures, although these associations have never been studied. An improved understanding of these associations is essential for advancing the knowledge base and informing healthcare strategies, particularly for fracture prevention and also reduction of hip fracture-related mortality in older adults. This study uses the 2018 China Health and Retirement Longitudinal Study (CHARLS) database, which contains a nationally representative sample of the Chinese population, to explore relationship between musculoskeletal pain and hip fractures. The study hypothesis is that the risk of hip fracture is associated with musculoskeletal pain at specific sites, as well as with the number of pain sites. The findings of this study enrich the current knowledge base and provide evidence from an ethnic population, which may be used as a point of comparison for future epidemiological studies.

## Methods

### Study population

This study analyzed data from the most recent follow-up questionnaire of CHARLS, which is a nationally representative longitudinal survey of the Chinese population conducted by the National School of Development of Peking University. The survey focuses on the middle-aged and elderly population in China, and includes interviews with Chinese residents aged 45 years or older and their spouses in their household. These interviews capture information about the respondents’ social, economic, and health status. The original CHARLS study was approved by the Ethical Review Committee of Peking University (approval number: IRB00001052-11015), and all data collection and analysis methods were conducted in accordance with relevant guidelines and regulations. Additionally, all respondents provided informed consent at the time of participation. A detailed description of CHARLS has been previously published [34].

**Fig. 1:**
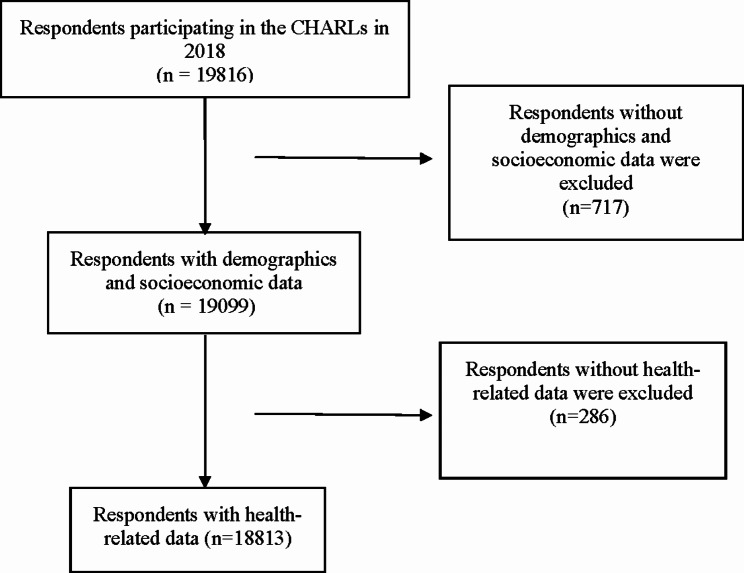
Data screening process for CHARLS data in 2018

The CHARLS database has been updated with follow-up surveys since the original study. Using the latest available CHARLS data from 2018, this study analyzed the risk factors of hip fractures, including individual factors and factors related to musculoskeletal pain. The inclusion criteria for the present study were: (1) individuals aged at least 45 years old in CHARLS 2018; (2) and having data regarding musculoskeletal pain. Exclusion criteria were: (1) missing data of demographics and medical information; (2) persons aged less than 45 years old; (3) missing data of musculoskeletal pain in CHARLS 2018; (4) persons without musculoskeletal pain. After data screening, 1003 respondents were excluded for missing data, a total of 18,813 respondents met the inclusion criteria for this study (Fig. 1).

### Data collection and preprocessing

The household interview collected information on demographic characteristics including gender, age, residential address, marital status, and employment status. Socioeconomic information, including education level and insurance status, was also recorded. Additionally, health-related behavior such as smoking and alcohol consumption were documented.

Regarding data collection for this study, participants were initially interviewed to determine if they experienced any physical pain. If the response was categorized as ‘a little’, ‘somewhat’, ‘quite a bit’, or ‘very’, participants were then asked to identify the specific body part(s) where they were currently experiencing pain. The participants were also inquired about any past instances of hip fracture.

Information collected from participants were categorized into groups based on their demographic characteristics. The musculoskeletal pain sites surveyed included the neck, shoulder, arm, wrist, fingers, back, waist, leg, knees, ankle, and toes. Some pain sites, such as the head, chest, and stomach, were excluded due to their association with visceral diseases. The number of pain sites was calculated based on the respondent’s answers regarding pain sites.

### Statistical analysis

This study utilized the mostly recently available 2018 CHARLS data to investigate the risk factors contributing to hip fractures. Univariate and multivariate analyses were conducted for different subgroups using chi-square tests and logistic regression. The regression models were performed stepwise, first adjusting for age, gender, residence, education, marital status, smoking, drinking, and employment status, and then further adjusting for musculoskeletal pain. P for trend test was performed to assess the trend of each continuous variable. The robustness and bias of parameter estimation were assessed using the bootstrap method. Restricted cubic spline regression was utilized to identify linear or non-linear relationships between variables. All data cleaning, processing, analysis, and calculations were carried out using R version 4.2.1. A *P* value of < 0.05 was considered statistically significant.

## Results

### Demographics data

Among the 19,816 participants from the 2018 CHARLS database, 18,813 respondents with relevant data were included in this study. The demographic characteristics of the included respondents are presented in Table 1. The mean age of respondents was 62 years, with a majority being female, married, and residing in rural areas. Almost half of the respondents had no formal education. The resident insurance coverage rate exceeded 90%.

**Table 1:** Characteristics of respondents in CHARLS in 2018

Demographics	Sample(n = 18,813)
Gender, n (%)	
Male	8980 (47.73)
Female	9833 (52.27)
Age, years, mean ± SD	62.14 ± 10.08
Age, group, years, n (%)	
45–54	5174 (27.52)
55–64	6252 (33.23)
65–74	5003 (26.59)
≥ 75	2384 (12.67)
Residence, n (%)	
Rural	14,029 (74.57)
Urban	4784 (25.43)
Education level, n (%)	
No formal education	8161 (43.38)
Elementary school	4161 (22.12)
Middle/high school	5670 (30.14)
College degree or higher	821 (4.36)
Marriage, n (%)	
Yes	16,015 (85.13)
No	2798(14.87)
Insurance status, n (%)	
No insurance	546 (2.90)
Basic medical insurance	17,240 (91.64)
Commercial insurance	164 (0.87)
Composite insurance	863 (4.59)
Smoking status, n (%)	
Yes	5086 (27.03)
Abstinence	2745 (14.59)
No	10,982 (58.37)
Drinking status, n (%)	
Yes	4937 (26.24)
Abstinence	702 (3.73)
No	13,174 (70.03)
Working status, n (%)	
Employed	11,982 (63.69)
Unemployed	6831 (36.31)

CHARLS, China Health and Retirement Longitudinal Study

### Relationship between hip fractures and anatomical site of musculoskeletal pain

**Fig. 2:**
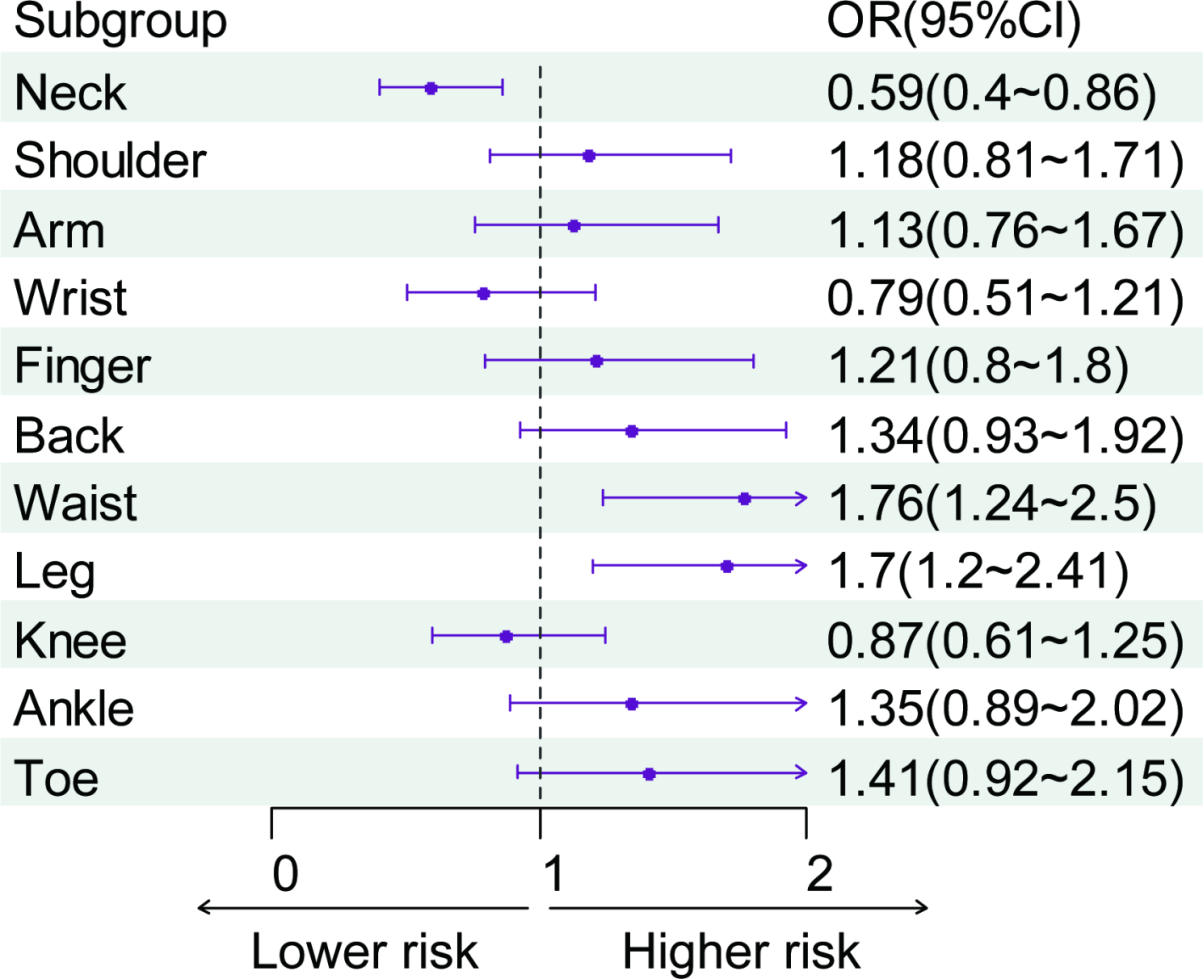
The multivariate analysis between risk of hip fractures and musculoskeletal pain

The results of correlation analysis between musculoskeletal pain and hip fractures is presented in Table 2. It was found that respondents experiencing waist pain and leg pain had a higher risk of hip fracture (*P* < 0.05). The correlation between pain sites and hip fractures is illustrated in Fig. 2.

**Table 2:** Statistics analysis of residents have ever fractured hip (n = 18,813)

Variables	Hip fracture		Univariate analysis			*P* trend	Multivariate analysis		
	Yes, n	No, n	OR	95CI%	*P*		OR	95CI%	*P*
Neck pain									
Yes	53	3392	1.47	(1.07,2.00)	**0.02**		0.59	(0.40,0.86)	**0.01**
No	162	15,206	ref	ref	ref		ref	ref	ref
Shoulder pain									
Yes	92	4892	2.10	(1.60,2.75)	**< 0.01**		1.18	(0.81,1.71)	0.38
No	123	13,706	ref	ref	ref		ref	ref	ref
Arm pain									
Yes	76	3730	2.18	(1.64,2.89)	**< 0.01**		1.13	(0.76,1.67)	0.55
No	139	14,868	ref	ref	ref		ref	ref	ref
Wrist pain									
Yes	50	2511	1.94	(1.41,2.67)	**< 0.01**		0.79	(0.51,1.21)	0.28
No	165	16,087	ref	ref	ref		ref	ref	ref
Fingers pain									
Yes	59	2688	2.24	(1.65,3.03)	**< 0.01**		1.21	(0.80,1.80)	0.36
No	156	15,910	ref	ref	ref		ref	ref	ref
Back pain									
Yes	78	3538	2.42	(1.83,3.21)	**< 0.01**		1.34	(0.93,1.92)	0.11
No	137	15,060	ref	ref	ref		ref	ref	ref
Waist pain									
Yes	131	6913	2.64	(2.00,3.47)	**< 0.01**		1.76	(1.24,2.50)	**< 0.01**
No	84	11,685	ref	ref	ref		ref	ref	ref
Leg pain									
Yes	103	4739	2.69	(2.05,3.52)	**< 0.01**		1.70	(1.20,2.41)	**< 0.01**
No	112	13,859	ref	ref	ref		ref	ref	ref
Knees pain									
Yes	95	5362	1.95	(1.49,2.56)	**< 0.01**		0.87	(0.61,1.25)	0.45
No	120	13,236	ref	ref	ref		ref	ref	ref
Ankle pain									
Yes	61	2474	2.58	(1.91,3.48)	**< 0.01**		1.35	(0.89,2.02)	0.15
No	154	16,124	ref	ref	ref		ref	ref	ref
Toes pain									
Yes	47	1691	2.80	(2.02,3.88)	**< 0.01**		1.41	(0.92,2.15)	0.11
No	168	16,907	ref	ref	ref		ref	ref	ref
The number of painful sites, n									
0	60	8407	ref	ref	ref	**< 0.01**			
1	18	2126	1.19	(0.70,2.01)	0.53				
2	20	1785	1.57	(0.94,2.61)	0.08				
3	10	1398	1.00	(0.51,1.96)	1.00				
4	11	1065	1.45	(0.76,2.76)	0.26				
5	20	921	3.04	(1.83,5.07)	**< 0.01**				
6	17	764	3.12	(1.81,5.37)	**< 0.01**				
7	17	582	4.09	(2.37,7.06)	**< 0.01**				
8	14	462	4.25	(2.36,7.65)	**< 0.01**				
9	10	417	3.36	(1.71,6.61)	**< 0.01**				
10	8	313	3.58	(1.70,7.55)	**< 0.01**				
11	10	358	3.91	(1.99,7.71)	**< 0.01**				

SD, Standard Deviation

### Relationship between hip fractures and the number of pain sites

**Fig. 3:**
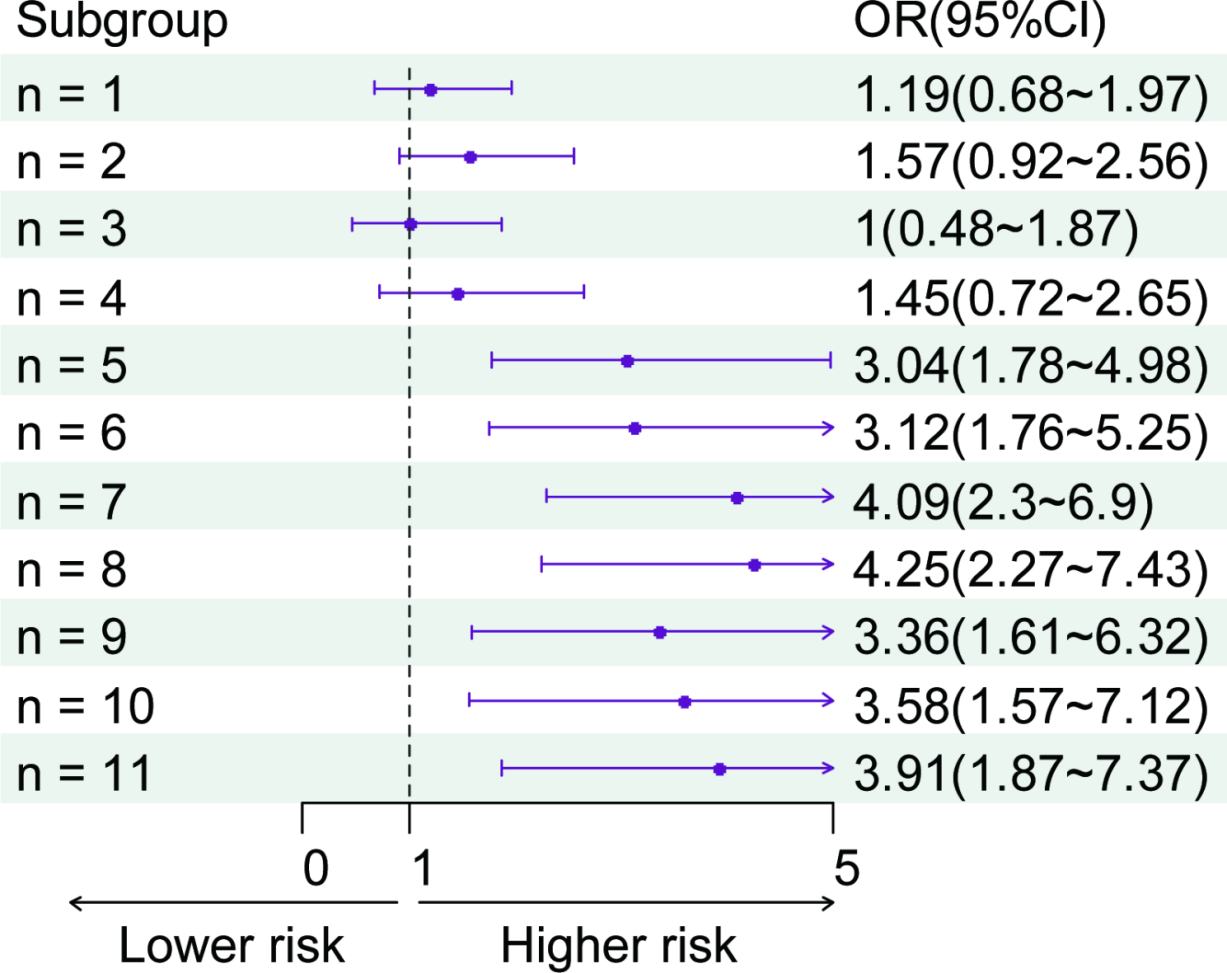
The multivariate analysis between risk of hip fractures and the number of painful sites

As shown in Table 2, when musculoskeletal pain occurs in more than 4 anatomical sites, an increased number of pain sites up to 8 total sites is significantly associated with a higher risk of hip fracture (*P* < 0.05). Furthermore, the correlation between the number of pain sites and hip fractures is depicted in Fig. 3.

### Relationship between hip fractures and other individual factors

**Fig. 4:**
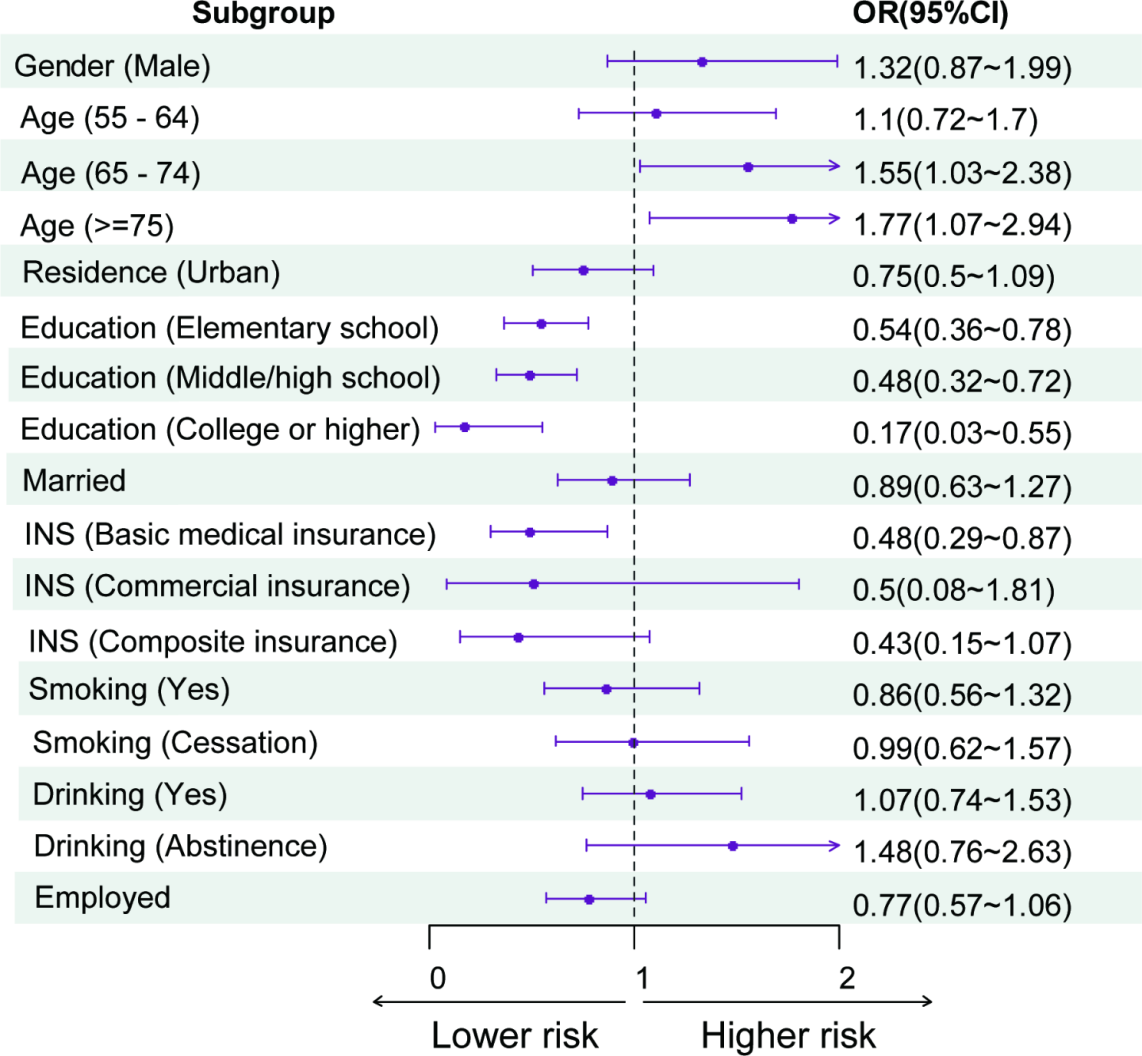
The multivariate analysis between demographic factors and risk of hip fractures

Among the 18,813 respondents, a total of 215 individuals reported that they have experienced a hip fracture. Table 3 presented the results of correlation analysis between various individual factors and hip fractures. The outcomes of multi-factor analysis are shown in Fig. 4. It was observed that among all non-pain-related individual factors, the occurrence of hip fractures was primarily associated with age and education level. Notably, the risk of hip fractures was found to increase with age among individuals aged 65 and above (*P* < 0.05). Additionally, respondents with lower education level exhibited a higher risk of hip fracture compared to those with higher education levels (*P* < 0.05). The correlation trend of both age and education level was tested to be statistically significant (*P* < 0.05). Moreover, the estimated regression coefficients of most variables had minimal bias, indicating a considerable overall robustness in their estimation. It was found that the association between age and fracture followed a linear relationship (Supplementary materials).

**Table 3:** Statistics analysis of residents have ever fractured hip (n = 18,813)

Variables	Hip fracture		Univariate analysis			*P* trend	Multivariate analysis			*P* trend
	Yes, n	No, n	OR	95%CI	*p*		OR	95%CI	*p*	
Gender										
Female	112	9721	ref	ref	ref		ref	ref	ref	
Male	103	8877	1.01	(0.77,1.32)	0.96		1.32	(0.87,1.99)	0.18	
Age										
45–54	37	5137	ref	ref	ref	**< 0.01**	ref	ref	ref	**0.01**
55–64	55	6197	1.23	(0.81,1.87)	0.33		1.10	(0.72,1.70)	0.65	
65–74	75	4928	2.11	(1.42,3.14)	**< 0.01**		1.55	(1.03,2.38)	**0.04**	
≥75	48	2336	2.85	(1.85,4.39)	**< 0.01**		1.77	(1.07,2.94)	**0.03**	
Residence										
Rural	179	13,850	ref	ref	ref		ref	ref	ref	
Urban	36	4748	0.59	(0.41,0.84)	**< 0.01**		0.75	(0.50,1.09)	0.15	
Education level										
No formal education	139	8022	ref	ref	ref	**< 0.01**	ref	ref	ref	**< 0.01**
Elementary school	36	4125	0.50	(0.35,0.73)	**< 0.01**		0.54	(0.36,0.78)	**< 0.01**	
Middle/high school	38	5632	0.39	(0.27,0.56)	**< 0.01**		0.48	(0.32,0.72)	**< 0.01**	
College degree or higher	2	819	0.14	(0.03,0.57)	**0.01**		0.17	(0.03,0.55)	**0.01**	
Marriage										
No	49	2749	ref	ref	ref		ref	ref	ref	
Yes	166	15,849	0.59	(0.43,0.81)	**< 0.01**		0.89	(0.63,1.27)	0.50	
Insurance status										
No insurance	15	531	ref	ref	ref		ref	ref	ref	
Basic medical insurance	192	17,048	0.40	(0.23,0.68)	**< 0.01**		0.48	(0.29,0.87)	**0.01**	
Commercial insurance	2	162	0.44	(0.10,1.93)	0.28		0.50	(0.08,1.81)	0.36	
Composite insurance	6	857	0.25	(0.10,0.64)	**< 0.01**		0.43	(0.15,1.07)	0.09	
Smoking status										
No	124	10,858	ref	ref	ref		ref	ref	ref	
Cessation	37	2708	1.20	(0.83,1.73)	0.34		0.99	(0.62,1.57)	0.96	
Yes	54	5032	0.94	(0.68,1.30)	0.70		0.86	(0.56,1.32)	0.48	
Drinking status										
No	151	13,023	ref	ref	ref		ref	ref	ref	
Abstinence	12	690	1.50	(0.83,2.71)	0.18		1.48	(0.76,2.63)	0.21	
Yes	52	4885	0.92	(0.67,1.26)	0.60		1.07	(0.74,1.53)	0.70	
Working status										
Unemployed	99	6732	ref	ref	ref		ref	ref	ref	
Employed	116	11,866	0.66	(0.51,0.87)	**< 0.01**		0.77	(0.57,1.06)	0.11	

SD, Standard Deviation

## Discussion

Hip fractures are associated with high mortality rates and significant costs, posing a major healthcare problem that also has devastating impacts on the individual, such as impaired physical function and quality of life particularly in the elderly [30, 35]. Factors contributing to an increased risk of hip fractures were of particular interest for investigation in this study as past studies in 2011 and 2013 using CHARLS data have indicated that China has a higher incidence of hip fractures compared to the United States and Japan [36, 37]. A recent report estimated that approximately 1,000 men and 1,000 women per 100,000 community residents in China experience hip fractures [38]. This study used the latest 2018 CHARLS data to analyze the risk factors contributing to the occurrence of hip fractures in the Chinese population. The findings suggested that an increased risk of hip fractures was associated with various individual factors of the respondents, the presence and location of musculoskeletal pain, and the number of pain sites. Gaining a better understanding of these influencing factors is crucial for preventing hip fractures among the elderly and reducing future mortality rates.

### Musculoskeletal pain as a factor influencing the risk of hip fractures

Although the association between musculoskeletal pain and hip fractures has been rarely explored, several studies have reported the relationship between musculoskeletal pain at different body sites and other types of fractures such as vertebral fracture [20, 31, 32]. This study confirmed a statistically significant risk of hip fracture associated with musculoskeletal pain in the waist and leg.

Both waist pain and leg pain have known impacts on central as well as lower limb proprioception, crucial for lower limb muscle strength and joint stability. These factors are positively correlated with falls risk and can contribute to the occurrence of hip fracture [39–41]. Therefore, for elderly individuals experiencing musculoskeletal pain, healthcare practitioners should pay particular attention to pain in the waist and leg, as these may play a greater role in hip fracture than pain at other body sites.

### Number of musculoskeletal pain sites and the risk of hip fractures

Previous studies have reported that individuals who experience pain at multiple sites, including the neck, back, hands, shoulders, hips, knees, and feet, are at increased risk of vertebral and non-vertebral (such as femoral, radial, ulnar, rib, and humeral) fractures [20]. However, hip fractures have been rarely reported in these studies due to their high mortality rate. Important to the knowledge base in this respect, the findings of this study suggest that the risk of hip fracture increases with the number of pain sites across 11 different areas in the body, namely the neck, back, waist, shoulders, arms, wrists, fingers, legs, knees, ankles, and toes. There is evidence that patients with multi-site pain have moderately elevated levels of systemic inflammation, reduced anti-inflammatory markers, and enhanced innate immunity [42–45]. These changes may induce a systemic reduction in bone strength due to bone remodeling from the effects of inflammation, thereby increasing the risk of fracture. Furthermore, other reports have reflected that an increase in the number of pain sites additively limits basic daily activities in the elderly [46–48]. This restriction of activity is often associated with multiple deleterious effects such as decline in muscle strength, balance ability, and physiological functions, as well as predisposition to or exacerbation of musculoskeletal diseases such as osteoporosis and osteoarthritis. These factors may individually or in combination increase the risk of falls in the elderly and also lead to an increased risk of hip fractures.

### Demographic factors influencing the risk of hip fractures

This study found that an increased risk of hip fractures was associated with a number of individual factors, which may provide new areas of focus when developing prevention strategies. Consistent with other studies [49–51], this study showed increased hip fracture risk with older age in people over 65 years. This association is likely due to declining physiological function with age, which may be a cause or consequence of decreases in physical activity, muscle strength, proprioception of the lower extremities, and physical reactive balance. These aging changes, together with an increased risk of osteoporotic fractures all increase the risk of hip fractures in older adults [52–54]. A particularly interesting finding from this study was that higher education levels were inversely associated with the risk of hip fracture. This may be partly due to the influence of education on individual behavior, such as elective levels of physical activity, nutritional supplementation, smoking, and alcohol consumption [55]. Some studies have also reported poorer outcomes for orthopedic patients who had received lower levels of education, manifested as higher pain scores, reduced range of motion, and poorer functional outcomes following intervention [56, 57].

### Study strengths and limitations

This study used the 2018 CHARLS data to conduct a detailed analysis of the risk factors associated with hip fractures in the Chinese population. The demographic characteristics of 18,813 participants were examined, 215 of whom had previously experienced a hip fracture. This incidence rate was consistent with findings from previous studies conducted within Chinese communities [38]. The study strengths arise from the ability to comprehensively analyze a large population sample, encompassing information on the participants’ demographics, pain data, and history of hip fractures. This comprehensive information enabled subgroup analyses based on age, sex, socioeconomic status, and health-related behavior, as well as trend tests to elucidate the factors contributing to increased hip fracture risk. The associations found to exist with different individual characteristics and pain sites constitute an important contribution to knowledge in the field, and serve as a point of comparison for future studies in other populations as well as a guide for devising prevention or intervention strategies.

This study has some limitations which may affect the interpretation of findings, primarily due to the nature of the CHARLS study from which data was drawn for analysis. Firstly, it is important to note that hip fractures assessment in CHARLS data relied on self-reporting and was not confirmed by clinical imaging, which could potentially lead to inaccuracies in the reported rate of fractures. Secondly, CHARLS data is focused on Chinese community residents aged 45 years and older, which may have led to an underestimation of hip fracture incidence since older age is associated with a higher risk of hip fractures. This factor may have impacted the analysis of associations with musculoskeletal pain. Thirdly, respondents with a greater number of pain sites may have better recollection and accuracy in reporting fractures compared to those with no or few pain sites. As respondents with multiple pain sites are a minority, the incidence of hip fractures could be underestimated in the sample used for this study, particularly if some of the respondents have experienced the fracture early in life but have forgotten about the experience at the time of the survey. Despite these limitations, the associations found between the risk of hip fractures and factors relating to the individual and musculoskeletal pain in this study provide valuable insights, and were drawn from a large and nationally representative sample of middle-aged and older adults, with important implications for public health and policy development.

## Conclusions

Based on data from the 2018 CHARLS survey, the risk of hip fractures was found to be associated with the location and number of body sites experiencing musculoskeletal pain, as well as with other non-pain-related individual factors. The findings of this study may be useful for guiding policy development, treatment strategies, and scientific or clinical study design.

### Electronic supplementary material

Below is the link to the electronic supplementary material.

**Supplementary Material 1: Sup Fig. 1** Histogram of bootstrapped samples of coefficients

**Supplementary Material 2: Sup Fig. 2** Restricted cubic spline graph of adjusted odds ratio between age and fracture

**Supplementary Material 3: Supplementary Table 1** Bootstrap statistics of all coefficients

## Data Availability

The datasets used and/or analyzed during the current study are available from the corresponding author on reasonable request. All data was from http://charls.pku.edu.cn/.
