# Supplementary figures and images for: Multidimensional characteristics of musculoskeletal pain and risk of hip fractures among elderly adults: the first longitudinal evidence from CHARLS
Source: BMC Musculoskelet Disord. 2024 Jan 2;25:4. doi: 10.1186/s12891-023-07132-z (PMC10759596; doi:10.1186/s12891-023-07132-z)

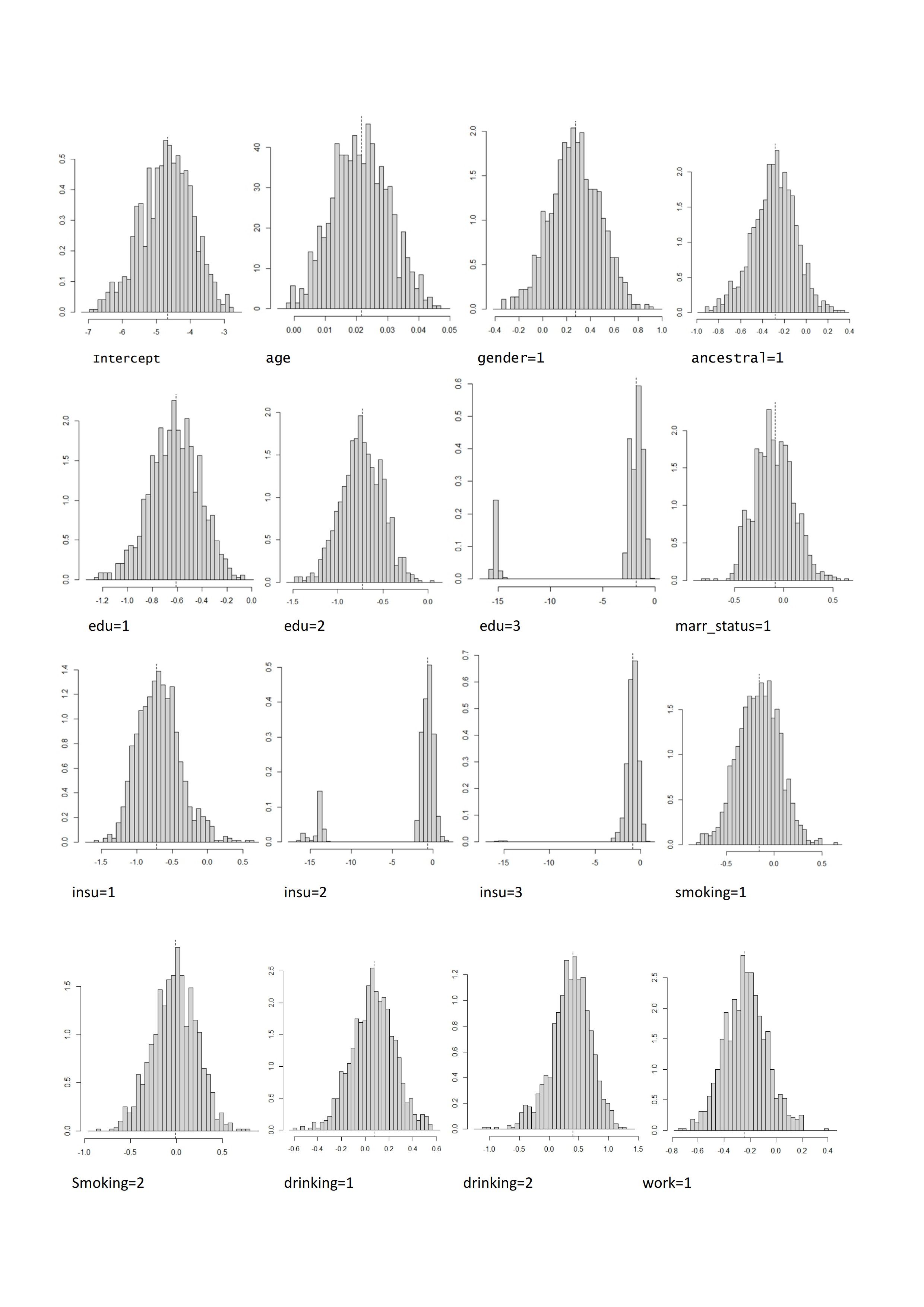

Supplement: Supplementary file 1 — Supplementary Material 1: Sup Fig. 1 Histogram of bootstrapped samples of coefficients [file 12891_2023_7132_MOESM1_ESM.png]

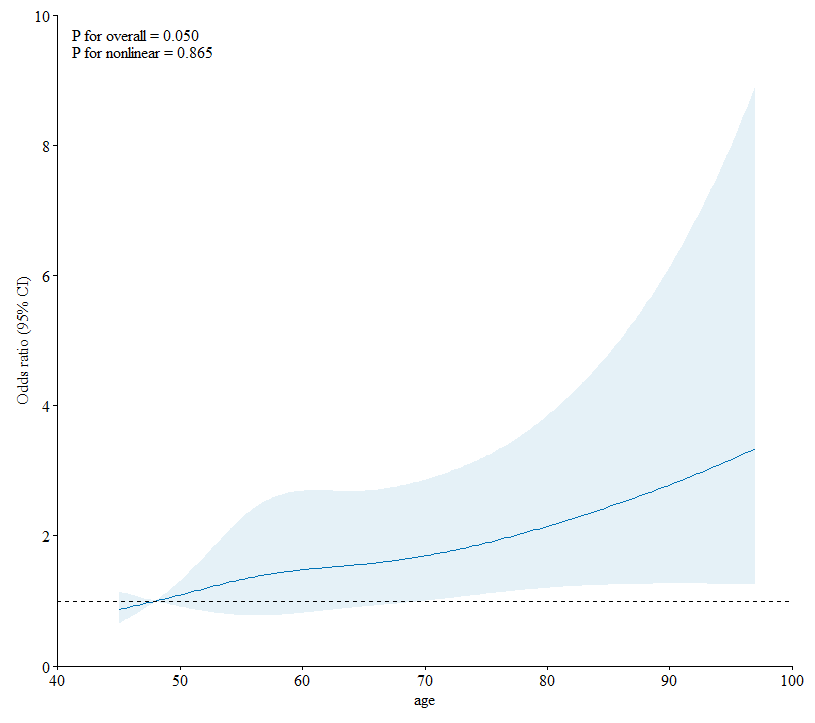

Supplement: Supplementary file 2 — Supplementary Material 2: Sup Fig. 2 Restricted cubic spline graph of adjusted odds ratio between age and fracture [file 12891_2023_7132_MOESM2_ESM.png]
