# Supplementary material for: Multidimensional characteristics of musculoskeletal pain and risk of hip fractures among elderly adults: the first longitudinal evidence from CHARLS
Source: BMC Musculoskelet Disord. 2024 Jan 2;25:4. doi: 10.1186/s12891-023-07132-z (PMC10759596; doi:10.1186/s12891-023-07132-z)
Supplement: Supplementary file 3 — Supplementary Material 3: Supplementary Table 1 Bootstrap statistics of all coefficients [file 12891_2023_7132_MOESM3_ESM.docx]

**Supplementary Table 1Bootstrap statistics of all coefficients**

| **Coefficients** | **Original** | **Bias** | **Std.error** |
| --- | --- | --- | --- |
| Intercept | -4.67 | -0.03 | 0.76 |
| Age | 0.02 | 0.00 | 0.01 |
| Gender=1 | 0.27 | 0.00 | 0.21 |
| Ancestral=1 | -0.28 | -0.01 | 0.20 |
| Edu=1 | -0.61 | -0.01 | 0.20 |
| Edu=2 | -0.73 | -0.01 | 0.23 |
| Edu=3 | -1.78 | -1.75 | 4.61 |
| Marr_status=1 | -0.09 | -0.01 | 0.20 |
| Insu=1 | -0.72 | 0.04 | 0.31 |
| Insu=2 | -0.68 | -1.68 | 4.53 |
| Insu=3 | -0.84 | -0.11 | 1.18 |
| Smoking=1 | -0.15 | -0.01 | 0.21 |
| Smoking=2 | -0.01 | -0.01 | 0.24 |
| Drinking=1 | 0.07 | -0.01 | 0.18 |
| Drinking=2 | 0.40 | -0.04 | 0.33 |
| Work=1 | -0.24 | 0.01 | 0.16 |

Edu, education；Insu, insurance;
